# Association of Small Fiber Function with Microvascular Perfusion of Peripheral Nerves in Patients with Type 2 Diabetes: Study using Quantitative Sensory Testing and Magnetic Resonance Neurography

**DOI:** 10.1007/s00062-023-01328-5

**Published:** 2023-08-07

**Authors:** Christoph M. Mooshage, Lukas Schimpfle, Zoltan Kender, Dimitrios Tsilingiris, Taraneh Aziz-Safaie, Anja Hohmann, Julia Szendroedi, Peter Nawroth, Volker Sturm, Sabine Heiland, Martin Bendszus, Stefan Kopf, Felix T. Kurz, Johann M. E. Jende

**Affiliations:** 1 Department of Neuroradiology, Heidelberg University Hospital, Im Neuenheimer Feld 400, 69120 Heidelberg, Germany; 2 Department of Endocrinology, Diabetology and Clinical Chemistry (Internal Medicine 1), Heidelberg University Hospital, Im Neuenheimer Feld 410, 69120 Heidelberg, Germany; 3 Department of Neuroradiology, Division of Experimental Radiology, Heidelberg, Germany; 4 Department of Neurology, Heidelberg University Hospital, Heidelberg, Germany; 5 German Center of Diabetes Research, associated partner in the DZD, Munich-Neuherberg, Germany; 6 Institute for Diabetes and Cancer (IDC), Helmholtz Diabetes Center, Helmholtz Center, Munich, Neuherberg, Munich, Germany; 7 Joint Heidelberg-IDC Translational Diabetes Program, Inner Medicine 1, Heidelberg University Hospital, Heidelberg, Germany; 8 German Cancer Research Center, Heidelberg, Germany

**Keywords:** Microangiopathy, Nerve perfusion, Diabetic neuropathy, Dynamic contrast enhanced imaging, Small fiber neuropathy

## Abstract

**Introduction/aims:**

Diabetic small fiber neuropathy (SFN) is caused by damage to thinly myelinated A‑fibers (δ) and unmyelinated C‑fibers. This study aimed to assess associations between quantitative sensory testing (QST) and parameters of peripheral nerve perfusion obtained from dynamic contrast enhanced (DCE) magnetic resonance neurography (MRN) in type 2 diabetes patients with and without SFN.

**Methods:**

A total of 18 patients with type 2 diabetes (T2D, 8 with SFN, 10 without SFN) and 10 healthy controls (HC) took part in this cross-sectional single-center study and underwent QST of the right leg and DCE-MRN of the right thigh with subsequent calculation of the sciatic nerve constant of capillary permeability (K^trans^), extravascular extracellular volume fraction (V_e_), and plasma volume fraction (V_p_).

**Results:**

The K^trans^ (HC 0.031 min^−1^ ± 0.009, T2D 0.043 min^−1^ ± 0.015; *p* = 0.033) and V_e_ (HC 1.2% ± 1.5, T2D: 4.1% ± 5.1; *p* = 0.027) were lower in T2D patients compared to controls. In T2D patients, compound z‑scores of thermal and mechanical detection correlated with K^trans^ (r = 0.73; *p* = 0.001, and r = 0.57; *p* = 0.018, respectively) and V_e_ (r = 0.67; *p* = 0.002, and r = 0.69; *p* = 0.003, respectively). Compound z‑scores of thermal pain and V_p_ (r = −0.57; *p* = 0.015) correlated negatively.

**Discussion:**

The findings suggest that parameters of peripheral nerve microcirculation are related to different symptoms in SFN: A reduced capillary permeability may result in a loss of function related to insufficient nutritional supply, whereas increased capillary permeability may be accompanied by painful symptoms related to a gain of function.

## Introduction

Diabetic neuropathy (DN) is one of the most common complications of diabetes mellitus with a lifetime prevalence of up to 50% [1]. Distal symmetric polyneuropathy (DPN), the most common type of DN, can be classified into three different subtypes based on the type of nerve fibers affected, namely small fiber neuropathy (SFN), large fiber neuropathy (LFN) and mixed fiber neuropathy (MFN) [1, 2]. Small fiber neuropathy is defined by selective or predominant damage of the thinly myelinated A (δ) and the unmyelinated C fibers [3], which is a typical feature of early stages of DN [4–7]. A problem regarding SFN is that it cannot be diagnosed via nerve conduction studies [3, 8]. Therefore, several methods have been established to diagnose SFN: the quantification of intraepidermal nerve fiber density (IENF) via skin biopsy is a well-established and standardized procedure that enables an objective analysis of the number of intraepidermal nerve fibers but is limited with respect to the analysis of fiber function [9]. Quantitative sensory testing (QST), a test battery of 13 sensory parameters, represents a standardized, non-invasive method that allows neurological deficits to be quantified [10–12]. The QST embodies a very sensitive method to diagnose SFN [8], especially with respect to measuring thermal thresholds [13]. For different sensory nerve fiber functions, QST allows detection of either a gain or a loss of function. A gain of function is defined as the pathologically increased/painful perception of a normally non-painful stimulus as in hyperalgesia/allodynia, whereas a loss of function is defined as a pathologically decreased perception of a sensory stimulus as in numbness [11]. Although QST poses a highly sophisticated method for the analysis of SFN, it only provides a limited insight into the underlying physiological changes of peripheral nerves. Changes in nerve perfusion, which have been hypothesized to pose a major contributor to both pain and structural nerve damage in DN, however, cannot be investigated through QST and other established clinical tools [14, 15]. Previous studies that combined high-resolution magnetic resonance neurography (MRN) with QST found associations between QST parameters and fascicular lesions of the sciatic nerve in patients with type 2 diabetes [16]. The finding of associations between MRN parameters of the proximal sciatic nerve and QST parameters obtained at a very distal level is supported by previous histological studies that found proximal sciatic demyelination to be associated with distal nerve fiber damage in diabetes [14].

Previous studies demonstrated that dynamic contrast enhanced (DCE) magnet resonance imaging (MRI) allows the assessment of peripheral nerve microcirculation [17]. Recent studies established DCI MRI of the sciatic nerve as a novel imaging biomarker in patients with type 2 diabetes [18–20]. It was found that a decrease of the sciatic nerve capillary permeability was associated with a decline of electrophysiologic parameters and an increase in clinical neuropathy scores, such as the neuropathy deficit score that allows diagnosis of LFN but not SFN [20]. In opposition to this, the results from preclinical studies suggest that an increased permeability of the blood-nerve barrier poses an important contributor to the development of painful DPN via an increased intraneural influx of cells and molecules [21–23].

This study combined DCE MRN and QST in healthy controls (HC) and patients with type 2 diabetes with and without SFN to assess potential associations between QST and microvascular nerve perfusion and to investigate whether capillary permeability is associated with symptoms of gain and loss of function of small fibers measured by QST.

## Material and Methods

### Study Design and Participants

This study was approved by the local ethics committee of (BLINDED) and was registered prior to the first assessments of participants (BLINDED, clinicaltrials.gov identifier NCT03022721). Screening and recruitment as well as clinical, serological, and electrophysiological examinations of all participants were conducted at the outpatient clinic of the department of internal medicine. Written informed consent was obtained by all participants. MRN and image processing was performed by the department of neuroradiology by investigators blinded to clinical data. As no randomization was conducted during recruitment of the study, the results may underlie effects of selection bias. Participants were sampled on the principle of convenience sampling: Patients and healthy controls were asked for their willingness to undergo DCE-MRN which was not mandatory to participate in the (BLINDED) study on diabetes complications (BLINDED). All participants who were either healthy controls (HC) or patients with type 2 diabetes and had undergone DCE-MRN, QST, electrophysiological and serological were potentially eligible for this study. Only participants who completed the entire study protocol underwent were included in the analyses. Groups were matched for age, sex, body mass index (BMI) and estimated glomerular filtration rate (eGFR) as these represent potential confounding factors of nerve perfusion in type 2 diabetes.

Overall, 28 study participants took part in this cross-sectional single-center study between June 2016 and May 2022 of whom 10 were HC (6 women, 4 men) and 18 were diagnosed with type 2 diabetes (9 women, 9 men). Of the type 2 diabetes patients 8 were diagnosed with SFN while 10 patients neither showed signs of SFN nor LFN (nDPN).

The overall exclusion criteria were as follows: age under 18 years, pregnancy or any contraindications for MRI or MR contrast agents, any history of myocardial infarction, coronary heart disease or heart surgery, spine surgery or lumbar disc extrusion as well as any risk factors for sarcopenia or neuropathy other than diabetes, such as malignant diseases, alcoholism, hypovitaminosis or any previous or ongoing exposure to neurotoxic agents. Also, we excluded patients with chronic conditions of the central nervous system, such as Parkinson’s disease, restless legs syndrome, or multiple sclerosis. To exclude severe renal insufficiency as a confounding factor and to minimize risks linked to the administration of contrast agent, patients with an eGFR of < 60 ml/min were excluded from this study. In addition, ongoing acute or chronic inflammatory disease were excluded through a detailed clinical and laboratory work-up.

### Electrophysiological and Serological Examinations

Blood was drawn from subjects in a fasting state followed by an immediate analysis by the central laboratory of Heidelberg University Hospital. The eGFR was calculated with the Chronic Kidney Disease Epidemiology Collaboration (CKD-EPI) formula [24].

All electrophysiological studies were conducted on the patients’ right leg by two specially trained medical technical assistants with more than 6 years of experience in electrophysiological assessments on patients with diabetes maintaining a skin temperature of 32 °C throughout the examination. The electrophysiological examination included the assessment of nerve conduction velocity (NCV) of tibial, peroneal, and sural nerves and sensory nerve action potential (SNAP) of the sural nerve.

### Quantitative Sensory Testing

A detailed medical history was obtained from every patient. A full QST was performed on the back of one foot by medical personnel trained and certified by the Department of Neurophysiology at the University Hospital of Mannheim. Cold detection threshold (CDT), warm detection threshold (WDT), cold pain threshold (CPT), heat pain threshold (HPT), thermal sensory limen (TSL), and paradoxical heat sensations (PHS) were determined on one back of the foot using a thermode (TSA-II; Medoc Ltd., Ramat Yishai, Israel) [10]. To test mechanical pain threshold (MPT), mechanical pain sensitivity (MPS), and wind-up ratio (WUR) a PinPrick stimulator set was used (MRC Systems GmbH, Heidelberg, Germany) while mechanical detection threshold (MDT) was determined using glas fiber von Frey filaments. A regular Q‑tip, a cotton ball, and a brush (SENSELab Brush-05; Somedic SenseLab AB, Sösdala, Sweden) were used to examine dynamic mechanic allodynia (DMA). To analyze the vibration detection threshold (VDT) a 64-Hz tuning fork was used. Pain threshold (PPT) was investigated with the use of a pressure algesiometer (FDN 200 with Rubber Tip 1 cm^2^; Wagner Instruments, Greenwich, CT, USA). Subsequently, the thermosensory functions of Aδ-fibers and C‑fibers are represented by CDT, WDT, and TSL while the nociceptive functions of Aδ-fibers and C‑fibers are evaluated through CPT, HPT, PPT, and especially MPS and MPT [10]. The MDT and VDT are used to assess the tactile functions of larger Aβ-fibers [10]. Conduction of QST has been described in detail previously [10, 25].

To put the parameters obtained into the context of an established reference cohort in order to determine whether participants suffered from SFN, each value was normalized to a published cohort of the same age, sex, and test region as issued by the German Research Network on Neuropathic Pain (Deutscher Forschungsverbund Neuropathischer Schmerz, DFNS) [10, 11, 26]:

$$\text{z-score}=(\mathrm{X}_{\text{patient}}-\mathrm{M}_{\text{controls}})/\mathrm{SD}_{\text{controls}}{,}$$

with X being the value of the respective parameter, M being the mean and SD being the standard deviation of the control group. The calculated z‑scores follow a normal distribution with a zero mean, unit variance and with scores of > 1.96 standard deviations (SD) and < −1.96 SD being outside the 95% confidence interval of relative reference data. Hereby, the former represents gain and the latter represents loss of functions of the respective parameter.

Next, single parameters of QST were clustered to create compound z‑scores as our main clinical outcome parameters as performed before: specifically, compound z‑score of thermal detection reflects sensory C‑fiber function (average values of CDT, WDT, and TSL), compound z‑score of thermal pain and (average values of CPT and HPT) of mechanical pain (MPT and MPS) reflect sensory Aδ-fiber function while compound z‑score of mechanical detection represents sensory Aβ-fiber function (VDT and MDT) [16].

As the aim of this study was to assess the impact of MRN perfusion parameters on SFN, we only included type 2 diabetes patients either without any signs of DN or solely with pure SFN. Thus, patients who had abnormal NCV or compound motor action potentials/sensory nerve action potentials in two of the three nerves assessed were regarded as patients with MFN or LFN and were excluded from this study. Nerve conduction studies were performed in accordance with the consensus criteria of the Normative Data Task Force [25]. Participants were assigned to the SFN group if the diagnostic criteria of “probable” or “definite” SFN [9] issued by the Toronto Diabetic Neuropathy Expert Group were met. A total of 8 participants were diagnosed with SFN of whom 3 met the criteria of “probable” and 5 of “definite” SFN, 10 participants showed no signs of neuropathy according to the criteria outlined above.

### MRN Imaging Protocol

High-resolution MRN in a 3.0 T MR-scanner (Magnetom Tim TRIO, Siemens Healthineers, Erlangen, Germany) was performed on every patient using a 15-channel transmit-receive extremity coil at the right thigh. The following MR sequences were applied centered to the sciatic nerve bifurcation at distal thigh level:

1. Axial high resolution T2-weighted turbo spin echo 2D sequence with spectral fat suppression. Repetition time = 5970 ms, echo time = 55 ms, field of view = 160 × 160 mm^2^, matrix size = 512 × 512, slice thickness = 4 mm, no interslice gap, voxel size = 0.3 × 0.3 × 4.0 mm^3^, 24 slices, 24 acquired images, total acquisition time = 4:42 min.
2. Axial T1-weighted volume interpolated breathhold examination (VIBE) sequence. Repetition time = 3.3 ms, echo time = 1.11 ms, field of view = 160 × 160 mm^2^, matrix size = 128 × 128, slice thickness = 4 mm, interslice gap = 0.8 mm, voxel size = 1.3 × 1.3 × 4.0 mm^3^; single acquisition at flip-angles of 5°, 8°, 11°, 14°, 17° (24 slices = 144 acquired images), total acquisition time = 30 s.
3. Axial T1-weighted VIBE sequence. Repetition time = 3.3 ms, echo time = 1.11 ms, field of view = 160 × 160 mm^2^, matrix size = 128 × 128, slice thickness = 4 mm, interslice gap = 0.8 mm, voxel size = 1.3 × 1.3 × 4.0 mm^3^, 50 repetitions (1200 acquired images) at a flip angle of 15°, contrast agent administration (Dotarem®, Guerbet, France, 0.1 mmol/kg, flow rate 3.5 ml/s) after completion of the sixth repetition, total acquisition time = 4:09 min.

### MRI Data Analysis

**Fig. 1:**
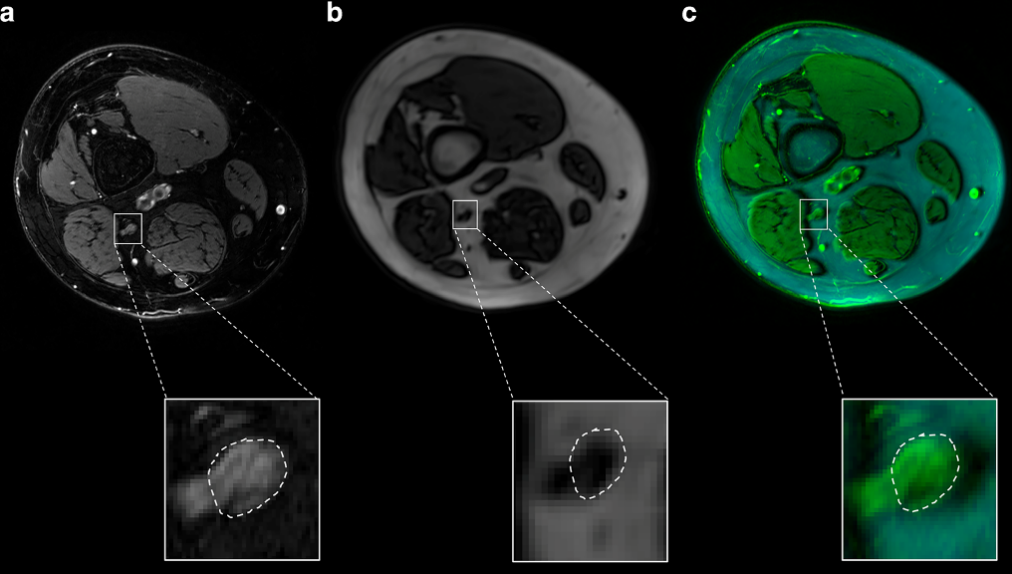
The process of coregistration of MRN sequences of a patient with T2D and small fiber neuropathy. An enlarged image of the sciatic nerve with encircled tibial compartment (*white dashed lines*) is shown highlighted by *white squares*. **a** Axial T2-weighted, fat-suppressed sequence at thigh level. No T2w-hyperintense or hypointense lesions, that would be a feature of large fiber neuropathy, can be seen in this T2D patient with SFN. **b** Axial T1-weighted VIBE sequence. **c** Coregistered image of T2-weighted (*green*) and VIBE (*cyan*) sequences

All images were evaluated for sufficient imaging quality and pseudonymized before image analysis. All MRN examinations were of sufficient quality. Two trained neuroradiologists with 2 and 6 years of experience in MRN manually segmented the sciatic nerve on the T2-weighted sequence using ImageJ [27]. A custom-written Matlab (MathWorks, Natick, MA, USA, R2020b) code was used for coregistration of the T1-VIBE sequence with affine transformations [28] as shown in Fig. 1. Arterial input function (AIF) was determined through a semimanual process as described in detail elsewhere [29]. Subsequently, the constant of volume transfer between plasma and the extravascular extracellular compartment (K^trans^), the volume fraction of extravascular extracellular space per unit volume of tissue (V_e_) and the blood plasma volume per unit volume of tissue (V_p_), which represent the main radiological outcome parameters, were calculated in accordance with the extended Tofts model with 𝐶𝑀(𝑡) representing the model tissue contrast agent concentration at time t [30]:

$$C_{M}(t)=K^{\text{trans}}\int^{t}AIF(t^{\prime})\exp(-\mathrm{K}^{\text{trans}[t-t^{\prime}]})dt^{\prime}+v_{p}AIF(t).$$

The extended Tofts model describes the distribution of injected MRI contrast agent in the two compartments of blood plasma and extravascular extracellular space. In this two-compartment exchange model, K^trans^ represents the constant of capillary permeability, whereas V_p_ and V_e_ represent the volume fractions of contrast agent in the blood plasma (V_p_) and in the extravascular extracellular space (V_e_) in relation to the total volume of the region of interest [31].

### Statistical Analysis

The MATLAB 7.14.0.0739 (R2012a, MathWorks Inc., Natick, MA, USA) and GraphPad Prism 7 (Grpahpad, San Diego, CA, USA) were used for all statistical analyses. Depending on data distribution, ordinary one-way ANOVA or Kruskal-Wallis test was used to compare groups and correction for multiple testing was performed with Tukey’s and Dunn’s multiple comparison tests. Likewise, Pearson or Spearman correlation coefficients were applied for correlation analyses. Partial correlation analyses were performed if multiple significant correlations were found for one parameter.

Based on previous studies on MRN imaging parameters in diabetes patients with and without DN [32] a Cohen’s d of 1.64 was calculated. For an alpha-level of 0.05 and a power of 0.9 a minimum of 16 participants (8 per group) was required for group comparisons.

## Results

### Participants

**Fig. 2:**
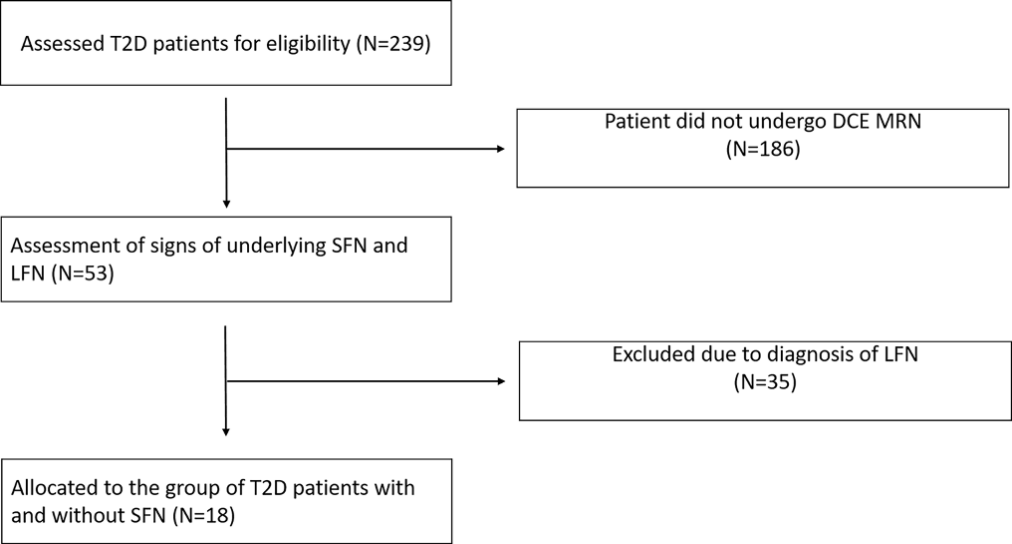
Flowchart of participants. *T2D* type 2 diabetes, *DCE MRN* dynamic contrast enhanced magnetic resonance neurography, *SFN* small fiber neuropathy, *LFN* large fiber neuropathy

In this study 239 patients with type 2 diabetes were assessed for eligibility. Of these, 53 patients were fully characterized through DCE-MRN, QST of the right foot as well as serological and electrophysiological testing and subsequently eligible for this study. Of these 35 were excluded from this study upon underlying diagnosis with LFN (Fig. 2).

A total of 124 HC were assessed for eligibility of whom 24 had undergone all required examinations to participate in this study. As both groups were matched for potential confounding factors 14 of these HC were excluded from this study.

### Clinical and Demographic Data

Of the type 2 diabetes patients 8 were diagnosed with SFN while 10 patients showed neither signs of SFN nor LFN (nDPN). No significant differences could be found for age, sex and BMI between HC and patients with type 2 diabetes or between patients with and without SFN. No differences could be found for compound z‑scores of thermal detection, thermal pain, mechanical detection and mechanical pain. Also, we did not find any difference in age, gender, BMI and duration of diabetes between SFN and nDPN patients.

The summary of all group comparisons on epidemiological, electrophysiological, serological and imaging data is provided in Tables 1 and 2.

**Table 1:** Group comparisons of demographic, serologic, electrophysiological, DCE perfusion parameters of the sciatic nerve and of compound z‑scores of quantitative sensory testing of all study participants

	HC	T2D	*p*-value
K^trans^ (min^−1^)	0.031 ± 0.009	0.043 ± 0.015	0.033^T^
Ve (%)	1.2 ± 1.5	4.1 ± 5.1	0.027^M^
Vp (%)	4.7 ± 0.5	5.3 ± 3.4	0.226^M^
Age (years)	59.9 ± 7.2	62.0 ± 10.1	0.567^T^
Gender	6w/4m	9w/9m	0.706^M^
BMI (kg/m^2^)	25.7 ± 3.6	28.3 ± 4.3	0.114^T^
Glucose (mg/dl)	92.9 ± 8.4	140.6 ± 39.9	<0.001^M^
HbA1c (mmol/mol)	36.9 ± 6.9	51.7 ± 13.9	<0.001^M^
HbA1c (%)	5.5 ± 0.6	6.9 ± 1.3	<0.001^M^
eGFR (ml/min)	91.1 ± 13.2	89.0 ± 17.1	0.832^M^
Sural nerve NCV (m/s)	44.4 ± 3.4	48.0 ± 7.9	0.189^T^
Sural nerve SNAP (µV)	10.6 ± 6.6	6.4 ± 3.9	0.053^T^
Peroneal nerve NCV (m/s)	45.5 ± 4.2	42.8 ± 5.3	0.180^T^
Peroneal nerve CMAP (mV)	7.64 ± 2.2	6.82 ± 4.36	0.338
Tibial nerve NCV (m/s)	45.2 ± 4.4	42.9 ± 5.6	0.270^T^
Tibial nerve CMAP (mV)	19.71 ± 5.99	14.59 ± 6.23	0.076^M^
Compound z‑score of thermal detection	−0.67 ± 0.54	−0.89 ± 1.17	0.579^T^
Compound z‑score of thermal pain	0.34 ± 0.66	−0.46 ± 0.82	0.708^T^
Compound z‑score of mechanical detection	−0.53 ± 1.30	−0.90 ± 1.61	0.543^T^
Compound z‑score of mechanical pain	0.99 ± 0.95	1.29 ± 0.57	0.296^T^

All values are displayed as mean ± standard deviation

*K*^*trans*^ constant of permeability, *v*_*p*_ plasma volume fraction, *v*_*e*_ extracellular extravascular volume fraction, *BMI* body mass index, *n.a.* not applicable, *eGFR* glomerular filtration rate, *NCV* nerve conduction velocity, *SNAP* sensory nerve action potential, *CMAP* compound motor action potential, *HbA1c* glycated hemoglobin

^M^* p* value obtained from Mann-Whitney U test

^T^* p* value obtained from t‑test

**Table 2:** Group comparisons of demographic, serologic, electrophysiological, DCE perfusion parameters of the sciatic nerve and of compound z‑scores of quantitative sensory testing of controls and T2D participants with and without SFN

	HC	nDPN	SFN	*p*-value	HC vs. nDPN	HC vs. SFN	nDPN vs. SFN
Ktrans (min^−1^)	0.031 ± 0.009	0.0474 ± 0.015	0.038 ± 0.153	0.034^K^	0.028	0.920	0.462
Ve (%)	1.2 ± 1.5	5.1 ± 5.4	2.8 ± 4.8	0.041^K^	0.034	0.754	0.649
Vp (%)	4.7 ± 0.5	4.5 ± 0.5	6.3 ± 5.1	0.450^K^	0.695	> 0.999	> 0.999
Age (years)	59.9 ± 7.2	64 ± 8.9	59.5 ± 11.50	0.405^K^	0.725	> 0.999	0.768
Gender	6w/4m	5w/5m	4w/4m	0.883^K^	> 0.999	> 0.999	> 0.999
BMI (kg/m^2^)	25.7 ± 3.6	28.9 ± 4.5	27.6 ± 4.225	0.240^A^	0.215	0.590	0.800
Glucose (mg/dl)	92.9 ± 8.4	140.3 + 33.6	140.9 ± 49.15	0.001^K^	0.003	0.009	> 0.999
HbA1c (mmol/mol)	36.9 ± 6.9	49.7 ± 9.2	54.1 ± 18.7	0.003^K^	0.003	0.009	> 0.999
HbA1c (%)	5.5 ± 0.6	6.7 ± 0.9	7.1 ± 1.7	0.003^K^	0.010	0.008	> 0.999
eGFR (ml/min)	91.1 ± 13.2	85.1 ± 18.9	93.8 ± 14.3	0.012^K^	> 0.999	0.708	0.228
Sural nerve NCV (m/s)	44.4 ± 3.4	47 ± 7.1	49.7 ± 9.7	0.424^K^	0.337	0.775	> 0.999
Sural nerve SNAP	10.6 ± 6.6	6.5 ± 4.2	6.4 ± 3.6	0.241^K^	0.396	0.543	> 0.999
Peroneal nerve NCV (m/s)	45.5 ± 4.2	42.6 ± 3.7	43.1 ± 7.0	0.405^A^	0.411	0.583	0.973
Tibial nerve NCV (m/s)	45.2 ± 4.4	43.0 ± 4.6	42.8 ± 6.9	0.548^A^	0.628	0.590	0.995
Compound z‑score of thermal detection	−0.67 ± 0.54	−0.46 ± 0.83	−1.43 ± 1.35	0.092^A^	0.866	0.214	0.088
Compound z‑score of thermal pain	0.34 ± 0.66	−0.37 ± 0.80	−0.57 ± 0.90	0.810^A^	0.997	0.817	0.856
Compound z‑score of mechanical detection	−0.53 ± 1.30	−0.63 ± 1.44	−1.29 ± 1.87	0.565^A^	0.989	0.572	0.652
Compound z‑score of mechanical pain	0.99 ± 0.95	1.301 ± 0.27	1.28 ± 0.83	0.584^A^	0.610	0.690	0.997

All values are displayed as mean ± standard deviation

*K*^*trans*^ constant of permeability, *v*_*p*_ plasma volume fraction, *v*_*e*_ extracellular extravascular volume fraction, *BMI* body mass index, *n.a.* not applicable, *eGFR* glomerular filtration rate, *NCV* nerve conduction velocity, *SNAP* sensory nerve action potential

^A^ *p* value obtained from ordinary one-way Anova and post hoc *p* values obtained from Tukeys multiple comparisons test

^K^ *p* value obtained from Kruskal-Wallis-test and post hoc *p* values obtained from Dunn’s multiple comparisons test

### Serological and Electrophysiological Parameters

No difference could be found for eGFR while HbA1c values were significantly higher in T2D patients with nDPN and SFN compared to HC (HC 36.9 mmol/mol ± 6.9, nDPN 49.7 mmol/mol ± 9.2, SFN 54.1 mmol/mol ± 18.7; *p* = 0.003 and *p* = 0.009).

No differences could be found for peroneal NCV, tibial NCV or sural NCV and SNAP.

### Perfusion Parameters

K^trans^ (HC 0.031 min^−1^ ± 0.009, SFN 0.038 min^−1^ ± 0.015, nDPN 0.0474 min^−1^ ± 0.015; *p* = 0.034) and V_e_ (HC 1.2% ± 1.5, nDPN 5.1% ± 5.4, SFN 2.8% ± 4.8, *p* = 0.041) were higher in nDPN patients compared to HC.

No differences could be found for V_p._

In HC we could not find any significant correlation of perfusion parameters. In the type 2 diabetes patient group K^trans^ correlated with V_e_ (r = 0.87, *p* < 0.001) which remained significant after partial controlled correlation analysis for age and BMI (r = 0.84, *p* < 0.001).

### Correlation Analysis of MR Perfusion Parameters and QST

In the type 2 diabetes patient group K^trans^ showed a negative correlation with age (r = −0.52, *p* = 0.026) while K^trans^ and V_e_ both showed a positive correlation with BMI (r = 0.59, *p* = 0.010 and r = 0.65, *p* = 0.003, respectively). In HC no significant correlations with age or BMI could be found.

**Fig. 3:**
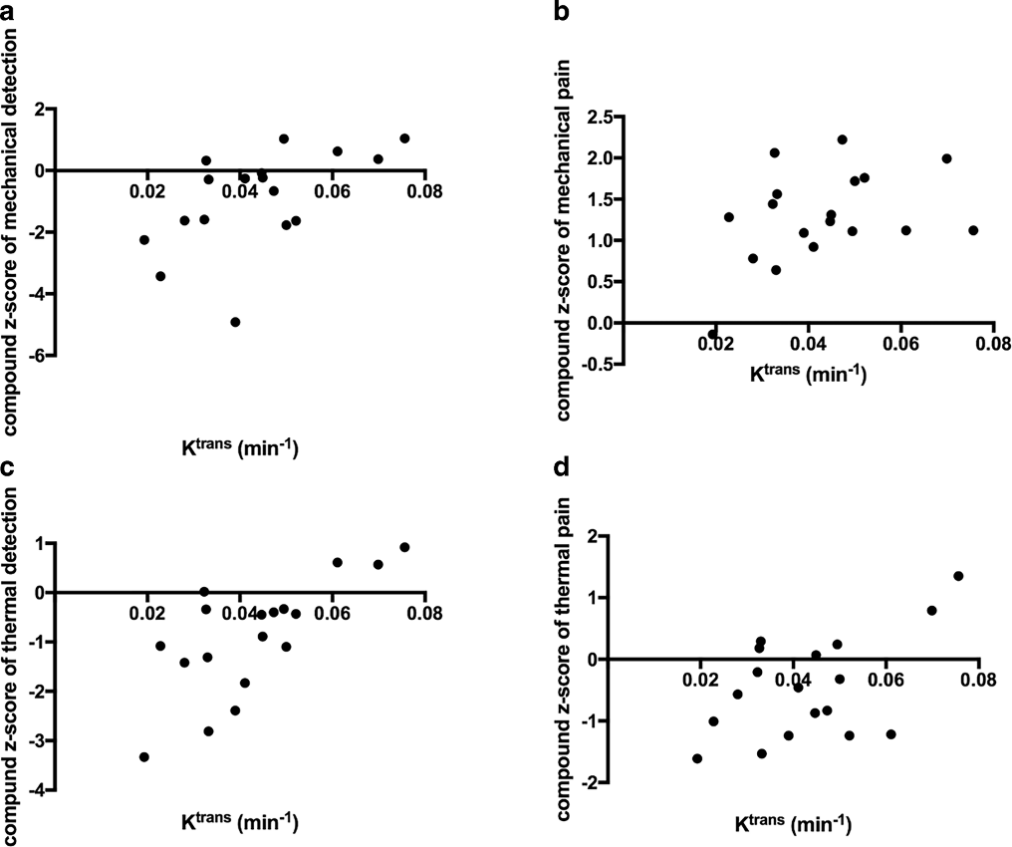
Correlations of the sciatic nerve constant of permeability (K^trans^) with compound z‑scores obtained from quantitative sensory testing of the right foot in patients with T2D. **a** Correlation of K^trans^ with compound z‑score of mechanical detection (r = 0.57, *p* = 0.018). **b** Correlation of K^trans^ with compound z‑score of mechanical pain (r = 0.39; *p* = 0.105). **c** Correlation of K^trans^ compound z‑score of thermal detection (r = 0.73, *p* = 0.001). **d** Correlation of K^trans^ and compound z‑score of thermal pain (r = 0.53, *p* = 0.024)

In type 2 diabetes patients the compound z‑score of mechanical detection correlated positively with K^trans^ (r = 0.57, *p* = 0.018; Fig. 3a), which did not reach statistical significance in partial correlation analysis controlled for age and BMI (r = 0.41, *p* = 0.133). Correlation of compound z‑score for mechanical pain with K^trans^ (r = 0.40, *p* = 0.105; Fig. 3b) did not reach a level of significance. For compound z‑scores of thermal detection we found a positive correlation with K^trans^ (r = 0.73, *p* = 0.001; Fig. 3c), which remained significant in partial controlled correlation analysis for age and BMI (r = 0.62, *p* = 0.010). Compound z‑scores of thermal pain correlated positively with K^trans^ (r = 0.53, *p* = 0.024; Fig. 3d), which did not reach a level of statistical significance in partial correlation analysis controlled for age and BMI (r = 0.44, *p* = 0.090). In HC K^trans^ correlated negatively with compound z‑scores of thermal pain (r = −0.64, *p* = 0.047). A summary of all correlations is provided in Table 3.

**Table 3:** Correlations of DCE MRN perfusion parameters with QST parameters of all T2D patients

	K^trans^		V_e_		V_p_	
	r	p	r	p	r	p
CDT	0.67	0.002^S^	0.75	< 0.001^S^	−0.26	0.307^S^
WDT	−0.59	0.009^S^	−0.66	0.003^S^	0.13	0.598^S^
TSL	−0.68	0.002^S^	−0.81	< 0.001^S^	0.22	0.372^S^
PHS	−0.15	0.584^P^	−0.02	0.925^S^	0.30	0.233^S^
CPT	−0.27	0.150^P^	0.43	0.073^S^	−0.40	0.103^S^
HPT	−0.15	0.544^S^	−0.29	0.249^S^	0.37	0.128^S^
MDT	−0.36	0.155^S^	−0.50	0.042^S^	0.35	0.174^S^
MPT	−0.34	0.173^S^	−0.38	0.117^S^	−0.15	0.549^S^
MPS	0.33	0.188^S^	0.32	0.200^S^	−0.40	0.101^S^
DMA	0.27	0.271^S^	0.12	0.634^S^	−0.43	0.072^S^
WUR	0.22	0.383^S^	0.23	0.351^S^	−0.20	0.416^S^
VDT	0.56	0.015^P^	0.65	0.004^S^	−0.33	0.183^S^
PPT	0.29	0.248^P^	−0.04	0.880^S^	−0.03	0.906^S^
z‑score CDT	0.69	0.001^P^	0.74	< 0.001^S^	−0.32	0.200^S^
z‑score WDT	0.62	0.006^P^	0.62	0.007^S^	−0.17	0.494^S^
z‑score TSL	0.72	0.001^P^	0.69	0.002^S^	−0.24	0.334^S^
z‑score CPT	0.34	0.166^P^	0.45	0.058^S^	−0.46	0.053^S^
z‑score HPT	0.15	0.541^S^	0.27	0.277^S^	−0.41	0.093^S^
z‑score MDT	0.46	0.064^P^	0.55	0.021^S^	−0.30	0.249^S^
z‑score MPT	0.27	0.276^P^	0.25	0.309^S^	−0.06	0.807^S^
z‑score MPS	0.40	0.098^P^	0.40	0.102^S^	−0.46	0.053^S^
z‑score WUR	0.06	0.822^P^	0.19	0.455^S^	−0.18	0.465^S^
z‑score VDT	0.56	0.016^S^	0.58	0.012^S^	−0.28	0.263^S^
z‑score PPT	−0.09	0.720^P^	−0.01	0.984^S^	0.09	0.726^S^
z‑score thermal detection	0.73	0.001^P^	0.67	0.002^S^	−0.30	0.219^S^
z‑score thermal pain	0.53	0.024^P^	0.43	0.078^S^	−0.57	0.015^S^
z‑score mechanical detection	0.57	0.018^P^	0.69	0.003^S^	−0.38	0.139^S^
z‑score mechanical pain	0.40	0.105^P^	0.47	0.051^S^	0.10	0.690^S^

All values are displayed as mean ± standard deviation

*CDT* cold detection threshold, *WDT* warm detection threshold, *TSL* thermal sensory limen, *CDT* cold detection threshold, *PHS* paradoxical heat sensations, *CPT* cold pain threshold, *HPT* heat pain threshold, *MDT* mechanical detection threshold, *MPT* mechanical pain threshold, *MPS* mechanical pain sensitivity, *DMA* dynamic mechanic allodynia, *WUR* wind-up ratio, *VDT* vibration detection threshold, *PPT* pressure pain threshold

^P^ *p* value obtained from Pearson correlation analysis

^S^ value obtained from Spearman correlation analysis

**Fig. 4:**
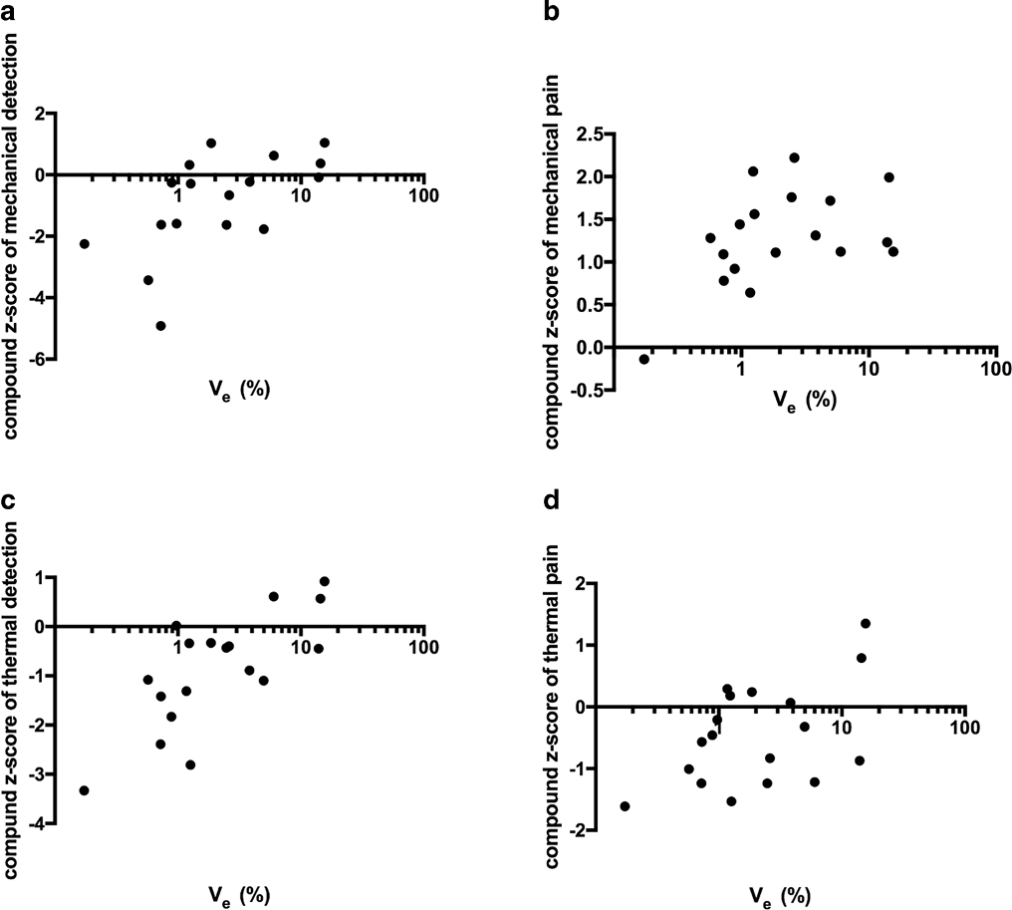
Correlations of the sciatic nerve extravascular, extracellular volume fraction (V_e_) with compound z‑scores obtained from quantitative sensory testing of the right foot in patients with T2D. **a** Correlation of V_e_ with compound z‑score of mechanical detection (r = 0.69, *p* = 0.003). **b** Correlation of V_e_ with compound z‑score of mechanical pain (r = 0.47, *p* = 0.051). **c** Correlation of V_e_ compound z‑score of thermal detection (r = 0.67, *p* = 0.002). **d** Correlation of V_e_ and compound z‑score of thermal pain (r = 0.43; p= 0.078)

In the T2D group, a positive correlation between Ve and compound z‑scores of mechanical detection (r = 0.69, *p* = 0.003; Fig. 4a) was found, which remained significant (r = 0.69, *p* = 0.004) in a partial correlation analysis controlled for age and BMI. A correlation of compound z‑scores of mechanical pain with V_e_ just fell short of reaching statistical significance (r = 0.47, *p* = 0.051; Fig. 4b) in a partial correlation analysis controlled for age and BMI (r = 0.48, *p* = 0.060). Compound z‑scores of thermal detection correlated positively with V_e_ (r = 0.67, *p* = 0.002; Fig. 4c) remaining significant in partial correlation analysis controlled for age and BMI (r = 0.63, *p* = 0.008). Furthermore, the correlation of V_e_ with compound z‑scores of thermal pain (r = 0.43, *p* = 0.078; Fig. 4d) as well as partial correlation analysis controlled for age and BMI (r = 0.47, *p* = 0.067) was not significant. Lastly, for V_p_ we could find a negative correlation with compound z‑scores of thermal pain (r = −0.57, *p* = 0.015) (Table 2). No such correlations were found between QST parameters and Ve or Vp in HC.

## Discussion

This study used QST and DCE 3T MRN to examine associations of parameters of SFN with peripheral nerve perfusion derived from DCE MRN in patients with type 2 diabetes. The main findings were: (i) in type 2 diabetes patients parameters of thermal and mechanical detection and of thermal pain correlated positively with the constant of capillary permeability (K^trans^) and the extravascular extracellular volume fraction (V_e_) of the contrast agent administered. (ii) In patients with type 2 diabetes compound z‑scores of thermal pain correlated negatively with the contrast agent plasma volume fraction (V_p_). (iii) In the type 2 diabetes patient group without SFN K^trans^ and V_e_ were higher compared to HC.

The correlations found for sciatic nerve capillary permeability (K^trans^) and extravascular extracellular volume fraction (V_e_) with compound z‑scores of thermal detection thresholds independent of confounding factors age and BMI indicate that a lower capillary permeability and extravascular extracellular volume fraction of peripheral nerves are linked to a loss of small fiber thermal sensory function, whereas an increase of peripheral nerve capillary permeability is associated with a gain of function. This is of particular interest with respect to the development of painful symptoms in DN. Preclinical studies have suggested that a breakdown of the blood-nerve barrier potentially contributes to pain in DN due to an intraneural leakage of inflammatory molecules and cells [22]. As many patients suffering from SFN initially experience painful symptoms related to a gain of function which is later followed by symptoms of a loss of function [33], one can assume that in affected patients initial symptoms are caused by a disruption of the blood-nerve barrier which is later followed by a decrease of capillary permeability due to processes associated with microangiopathy in type 2 diabetes, such as endothelial damage and perivascular fibrosis [34]. This assumption is further supported by the finding that in our cohort capillary permeability was higher in patients with type 2 diabetes diagnosed without SFN compared to HC, while previous studies on LFN found nerve permeability to be decreased in type 2 diabetes LFN [20]. As SFN is regarded to be an early finding in patients with distorted glucose metabolism [4, 35], similar to the disruption and subsequent increased permeability of the blood nerve barrier [36], we hypothesize that an increase in capillary nerve permeability in type 2 diabetes patients may result in painful SFN which, once capillary permeability decreases, might turn into a loss of function in SFN and is ultimately accompanied by LFN. This assumption might also explain why there was no difference in perfusion parameters between type 2 diabetes patients with and without SFN: The prevalence of both increased and decreased K^trans^ values in the SFN group may have resulted in a counterbalance of K^trans^. This hypothesis, however, requires longitudinal data to be verified.

One may of course argue that z‑scores of QST parameters did not differ significantly between T2D patients with and without SFN and that, therefore, patients were not diagnosed correctly. It is important to bear in mind, however, that the SFN group comprised patients with either pathologically elevated or decreased z‑scores of different QST parameters. Therefore, it is not surprising that results do not significantly differ from participants without SFN.

One may of course question whether parameters of the sciatic nerve microcirculation are causally related to QST parameters measured far more distally at the level of the foot. Interestingly, previous studies have found that SFN in patients with diabetes does not appears to be dependent on length [37] and that in diabetic polyneuropathy proximally conducted NCS correlates with intraepidermal neve fiber density [38]. In the light of histological studies that found proximal demyelination in DN to be related to nerve ischemia and to be associated with fiber loss at distal levels [14, 39, 40], it seems justified to assume that there is a causal relationship between parameters of proximal nerve perfusion and distal QST.

This study has several limitations: First, the cross-sectional design does not allow conclusions to be drawn on the longitudinal and causal association of nerve perfusion and QST. Also, the small size did not allow correlation analyses to be carried out separately for patients without SFN and patients with pure SFN. It should be taken into account, however, that the aim of this study was not to investigate differences between patients with and without SFN, but to assess the impact of peripheral nerve perfusion on small fiber function in type 2 diabetes. As patients with LFN were excluded from this study based on pathologic nerve conduction studies, our patient cohort represents a well-defined group of type 2 diabetes patients without relevant large fiber damage, which allows to solely attribute the measurements of QST to small fiber damage. Yet, it has to be considered that nerve fiber damage represents a continuous spectrum in patients with T2D [4, 16] with SFN and LFN most likely occurring simultaneously. The prevalence of either diagnosis strongly depends on the predominance of symptoms and the diagnostic criteria applied [2]. Furthermore, it has been shown before that peripheral nerve damage in type 2 diabetes represents a continuous process from subclinical nerve lesions to symptomatic nerve damage [16], while this study found QST parameters to be correlated with parameters of nerve perfusion in a cohort comprised of type 2 diabetes patients with and without SFN. As a consequence, we believe it is justified to assume that our findings of a mixed cohort of SFN patients and patients without DN accurately represent the impact of nerve perfusion on QST parameters at an early stage of DN more accurately than comparing groups of patients with and without SFN. One may also argue that sural nerve SNAPs were lower in the T2D group compared to controls and that, therefore, LFN was already present in the T2D group. It should be considered, however, that SNAPs in the T2D group were still within the range of normal values defined by previous studies [41, 42], which renders the assumption that LFN is a major contributor to the results obtained as unlikely. Another important limitation of this study is the sample size of type 2 diabetes patients and controls, which does not allow all potential confounders for the observed differences and correlations to be ruled out. Yet, it should be noted that patients and controls were matched for age, BMI, gender, and renal function to minimize confounding. Also, we conducted partial controlled correlation analysis for age and BMI as main confounders of K^trans^ and V_e,_ as outlined above. The study is further limited by the fact that only a relatively short segment of the sciatic nerve of approximately 10 cm was investigated. It has been shown by previous studies, however, that the examined ROI at the level of the sciatic nerve bifurcation is the region of predominant nerve fiber damage in patients with diabetic neuropathy and that structural and functional nerve damage detected in this region reflects nerve function further distally [43].

In summary, our study is the first to find correlations of QST parameters with parameters of peripheral nerve perfusion obtained from DCE MRN. The results indicate that changes in nerve perfusion due to microangiopathy pose a relevant contributor to SFN in type 2 diabetes. Longitudinal studies with a higher number of patients, especially ones underlying symptoms of gain of function, will be needed to verify whether an increased capillary permeability is associated with gain and a decreased capillary with a loss of function in patients with SFN.

## Abbreviations

CDT: Cold detection threshold
CPT: Cold pain threshold
DCE: Dynamic contrast enhanced
DMA: Dynamic mechanical allodynia
DN: Diabetic neuropathy
DPN: Distal symmetric neuropathy
HC: Healthy controls
HPT: Heat pain threshold
K^trans^: Constant of volume transfer between plasma and the extravascular extracellular compartment
LFN: Large fiber neuropathy
MDT: Mechanical detection threshold
MPS: Mechanical pain sensitivity
MPT: Mechanical pain threshold
MRN: Magnetic resonance neurography
NCV: Nerve conduction velocity
nDPN: Patients without signs of large or small fiber neuropathy
PHS: Paradoxical heat sensations
PPT: Pain threshold
QST: Quantitative sensory testing
SFN: Small fiber neuropathy
SNAP: Sensory nerve action potential
TSL: Thermal sensory limen
V_e_: Volume fraction of extravascular extracellular space per unit volume of tissue
V_p_: Blood plasma volume per unit volume of tissue
VIBE: Volume interpolated breath hold examination
VDT: Vibration detection threshold
WDT: Warm detection threshold
WUR: Wind-up ratio
